# Cognitive deficits for facial emotions among male adolescent delinquents with conduct disorder

**DOI:** 10.3389/fpsyt.2022.937754

**Published:** 2022-08-23

**Authors:** Hui Kou, Wei Luo, Xue Li, Ye Yang, Min Xiong, Boyao Shao, Qinhong Xie, Taiyong Bi

**Affiliations:** ^1^Center for Mental Health Research in School of Management, Zunyi Medical University, Zunyi, China; ^2^The Institute of Ethnology and Anthropology, Chinese Academy of Social Sciences, Beijing, China; ^3^School of Criminal Justice, China University of Political Science and Law, Beijing, China; ^4^Psychological Guidance Center, Zunyi Medical University, Zunyi, China

**Keywords:** conduct disorder, male adolescent delinquents, attentional bias, working memory, facial expressions

## Abstract

According to the social-cognitive theory and the social-information-processing theory, individuals with conduct disorder, a persistent and repetitive pattern of problematic behavior, might have cognitive biases toward hostile facial expressions. However, according to the optimal stimulation/arousal theory, the stimulation-seeking theory and the fearlessness theory, individuals with conduct disorder might have less fear and show less response to hostile or threatening facial expressions. To reconcile the discrepancy, we examined the cognitive biases including attentional processing and working memory processing to emotional faces among adolescents with conduct disorder. 35 male adolescent delinquents with conduct disorder and 35 age-matched delinquents without conduct disorder completed a visual search task and a delayed-match-to-sample task to examine their attentional processing and working memory processing for sad, angry, happy, and fearful faces, respectively. It was found that conduct disordered individuals searched angry and fearful faces, rather than sad and happy faces, more slowly than individuals without conduct disorder. However, no difference in mnemonic processing for facial emotions was found between groups. The results indicated that male adolescent delinquents with conduct disorder showed deficits in attentional orientation to hostile and threatening faces, supporting the optimal stimulation/arousal theory, the stimulation-seeking theory and the fearlessness theory, but not the social-cognitive theory.

## Introduction

Conduct disorder (CD) is a highly impairing psychiatric disorder that usually manifests in childhood or adolescence. It is defined as a persistent and repetitive pattern of problematic behavior that violates others’ rights or that violates age-appropriate social norms or rules, and is characterized by a pattern of severe antisocial and aggressive behavior (1). CD is a psychiatric disorder associated with delinquency or crime. Previous studies revealed that delinquent or criminal youth were more likely to show CD symptoms or had more severe CD symptoms (2, 3). Additionally, CD could predict future antisocial outcomes and increase the risk of future crime (4–6).

Aggression may be developed and shaped by the manners that humans percept, process, store and retrieve information (7). According to the social-cognitive theory and the social-information-processing theory, the schema is the knowledge structure shaped by one’s unique experience and can automatically guide his/her social information processing (8–11). Previous studies revealed that aggressive or violent individuals were more likely to hold or endorse aggression-related schema (12–14). They usually showed a pattern of biased processing, such as the negative social-cognitive bias (15), the attentional bias toward hostile social cues (16–18), selective recall for hostile social cues (19), and the hostile attribution bias (19, 20). Moreover, attention to antisocial semantic cues could predict high aggression (21). Regarding CD individuals, as they often experience exposure to childhood abuse or maltreatment (22–26) and exposure to aggressive/deviant models or peers (27–29), they may develop a maladapted or distorted schema, which leads them to preferentially encode hostile cues, to mentally represent social cues as threats, to easily access aggressive responses and finally to engage in aggressive behavior.

Among social cues, facial expressions are one of the most frequently encountered in daily life and most effective in conveying one’s emotions and hostility. For example, an angry face indicates hostility and aggression, while a sad face implies the need for help and social support. CD individuals may thus develop biased cognitive processing on emotional faces. Behavioral studies demonstrated that 7- to 13-year-old children with CD interpreted emotions less accurately than controls and tended to misinterpret emotions as anger (30), and adolescents with CD were more likely to confuse fear with anger relative to healthy controls (31). Another study revealed that children with CD showed a stronger association between the hostile attribution bias and the attentional bias to angry faces compared with controls (32). On the neurophysiological level, CD showed stronger mismatch negativity (MMN) induced by fearful rather than sad syllables in an auditory oddball paradigm (33). A recent study revealed that aggressive males showed selectively attentional bias to angry faces, and undifferentiated P3 amplitude between angry and neutral faces (34). Another study found that aggression was associated with enhanced amygdala reactivity to angry faces (21). Taken together, evidence suggested that individuals with CD held the aggressive or hostile schema and may thus show biased processing of hostile or threatening information.

However, other perspectives support that CD may show reduced response or avoidance to hostile or threatening information. According to the optimal stimulation/arousal theory (35–37), the stimulation-seeking theory (38, 39), and the fearlessness theory (40, 41), individuals with CD have a lower level of physical arousal which reflects less fear and thus seek exaggerated external stimulation to lead their physical activity to reach the optimal level, and in turn show less response to affective information. Researchers observed a lower level of emotional response to unpleasant slides (42) and reduced corrugator muscle response to angry faces among CDs (43). Neuroimaging evidence revealed reduced activations in the amygdala to negative pictures or angry faces among CDs (44–46). Furthermore, studies also revealed that adolescents with CD showed attentional avoidance and difficulty in attentional disengagement from facial expressions including angry, fearful and happy faces compared to controls (47). An eye-movement tracking study revealed that adolescents with CD fixated less on fearful and sad faces (48). Therefore, it is still in debate whether individuals with CD show larger or less cognitive bias to emotional stimuli, especially to hostile or threatening faces.

To reconcile the discrepancy among previous studies and perspectives, we examined the cognitive biases including attentional processing and working memory processing to emotional faces among CD adolescents by adopting classical paradigms. Dot-probe paradigm (47) and emotional stroop paradigm (32) have been adopted to assess attentional processing of emotional faces among individuals with CD. The results were mixed. Compared to controls, adolescents with CD showed attention bias to facial expressions in a dot-probe task (47), while children with CD did not show any attentional biases to facial expressions in a pictorial emotional Stroop task (32). In the present study, we adopted the visual search paradigm which has a high ecological validity as it mimics everyday situations in which one attempts to find a target face among distractive faces. In this task, participants were asked to detect, locate or identify the target among distractors as quickly as possible and the results may reveal how attention suppresses irrelevant distractors as well as shifts/orients attention to the target. The performance in the visual search task could be modulated by facial emotions. Some researchers found a superiority effect on angry faces (49–53), while others found a superiority effect on happy faces (54–56). Nevertheless, the visual search paradigm is an effective and stable task to reveal the visual attentional processing of facial emotions. Relative to attentional processing, visual working memory processing on emotional faces among individuals with CD has been largely known. Working memory, which is a fundamental cognitive function of human, is usually characterized as the ability to maintain and manipulate perceptual information in a short period of time (57). Three subsystems were identified, including a central executive system to process information, and two slave systems of visuospatial sketch pad and phonological loop to store visual and verbal information, respectively. N-back task has been used to assess the working memory for object (e.g., letter) and spatial position among individuals with CD (58–60), which revealed that CD group performed worse than controls did, and CD symptoms were correlated with reduced P3 amplitude in the context of low working memory load. However, n-back task mainly reveals the updating mechanism in working memory processing and some researchers indicate its inefficiency in measuring working memory (61). In the present study, we adopted the delayed-match-to-sample (DMTS) paradigm which is one of the most common tasks to study visual working memory. The DMTS task consists of three phases, including a sample (encoding) phase, a delay (maintenance) phase, and a test (retrieval) phase. It is mainly used to examine the accuracy and capacity in encoding and maintaining visual stimuli. Previous studies showed stable test-retest reliability in DMTS task (62) and stable brain structures associated with the task (i.e., dorsolateral prefrontal cortex, fusiform gyrus and posterior parietal cortex) (63). Taken together, if the social-cognitive theory and the social-information-processing theory are critical mechanisms in the development of CD, we may predict that CD adolescents show higher attentional and working memory biases to frightening and hostile faces; if the optimal stimulation/arousal theory, the stimulation-seeking theory and the fearlessness theory are critical mechanisms, we may predict the opposite trend, that is, CD adolescents show lower attentional and working memory biases to threatening and hostile faces.

## Materials and methods

### Participants

Male adolescent delinquents in a reform school and a reformatory in Guizhou province of China underwent a structured clinical interview with the Diagnostic and Statistical Manual of Mental Disorders from DSM-IV-TR Axis I Disorders (64). The diagnosis of CD and its severity was established on this 15-item screening questionnaire which consists of four factors, including cruelty to humans and animals (e.g., bullying, fighting, and physical injury to pets), destruction of property (e.g., arson and vandalism), deception or theft (e.g., lying for self-interest), and serious violations (e.g., playing truant). If individuals who meet three of these criteria in one year and meanwhile meet at least one criterion within half a year are diagnosed to have conduct disorder. Furthermore, individuals meeting five or more criteria were assigned a “moderate-to-severe” conduct disorder diagnosis (65).

We performed a power analysis to determine the sample size using the G-Power 3.1.9.7 software (66). In order to find a significant interaction effect between emotion and group, at the level of η*_*p*_*^2^ = 0.1, α = 0.05, power = 0.95, the required total sample size is 22. We recruit 35 subjects for both groups, resulting in a total sample size of 70. 35 delinquents who met five or more criteria were assigned to the CD group. And 35 age-matched delinquents, who didn’t meet three of these criteria in one year nor meet at least one criterion within half a year, were assigned to the non-CD group. No significant difference in age was found between CD (*M* = 16.51, *SD* = 1.46, range from13 to 20) and non-CD (*M* = 16.80, *SD* = 1.37, range from 13 to 20) groups (*t* = 0.84, df = 68, *p* = 0.402). All participants are right-handed and their vision or corrected vision is normal. Exclusion criteria included a history of mental illnesses or a family member’s history of mental illness. All procedures performed in this study involving human participants were in accordance with the ethical standards of the Ethical Committee of Human Research at a medical university and with the 1964 Helsinki declaration and its later amendments or comparable ethical standards. Written informed consent was given by all adolescent delinquents and their legal guardians. All the participants completed the visual search experiment. However, three delinquents in the CD group quit the DMTS experiment for personal reasons. The order of the tasks was randomized across subjects.

### Measurements

The Inventory of Callous-Unemotional Traits (ICU) The 24-item scale consists of three subscales including callousness, uncaring and unemotional traits (67, 68). Callousness refers to the callous attitude toward others, uncaring is characterized by the lack of caring about performance and the unemotional trait is characterized by the lack of emotional expression. Response options range from 0 = not at all true to 3 = definitely true. Higher scores indicate a higher level of CU traits.

The Aggression Questionnaire (AQ) The 29-items scale can be used to assess aggression and consists of four factors: physical aggression, verbal aggression, anger, and hostility (69). Participants rate each item on a Likert scale from 1 = extremely uncharacteristic of me to 5 = extremely characteristic of me. Higher scores indicate a higher level of aggression.

The Short Form of the Childhood Trauma Questionnaire (CTQ-SF) This version of the CTQ contains 28 items (25 clinical items and 3 validity items) assessing childhood maltreatment, which consists of five factors: emotional abuse, physical abuse, sexual abuse, emotional neglect and physical neglect (70). Each item is rated on a Likert scale from 1 = never to 5 = always. A higher score implies more frequent exposure to maltreatment in childhood.

The Self-Control Scale (SCS) This 36-items scale consists of five subscales: self-discipline, deliberate/non-impulsive action, healthy habits, work ethic, and reliability (71). Participants rate each item on a 5-point Likert scale ranging from 1 = not at all like me to 5 = very much like me. The Chinese version of the SCS consists of five factors: impulse control, resistance to temptation, healthy habits, concentration on work and abstinence from entertainment. Confirmatory factor analysis (CFA) indicated a good construct validity for the revised SCS (72). Higher scores indicate better self-control.

The Moral Disengagement Scale (MDS) This 32-item scale consists of eight factors, including euphemistic labeling, distortion and minimization of consequences, moral justification, diffusion of responsibility, displacement of responsibility, disadvantageous comparisons, dehumanization, and victim-blaming (73, 74). Participants rate each item on a Likert scale from 1 = extremely disagree to 5 = extremely agree. Higher scores suggest a higher level of moral disengagement.

### Visual search task

#### Stimuli

The experimental stimuli were selected from the Chinese Affective Picture System (CAPS). Search targets included 16 sad faces, 16 angry faces, 16 happy faces, and 16 fearful faces, half of which were female faces. 58 neutral faces (29 female faces) were selected as distractors. All pictures were grayscaled and an oval mask was used to remove non-facial features (e.g., hair, neck, ears) from each face. Then, all the images were cropped into a uniform size (130 × 150 pixels), and the brightness and contrast were matched. A repeated-measures ANOVA found that the valences were significantly different among categories [*F*(3, 45) = 294.80, *p* < 0.001, η*_*p*_*^2^ = 0.952). *Post-hoc* tests suggested that the valence of happy faces (*M* = 6.75, *SD* = 0.51) was significantly higher than angry (*M* = 2.62, *SD* = 0.40), sad (*M* = 2.84, *SD* = 0.54), and fearful (*M* = 2.80, *SD* = 0.42) faces (*p*s < 0.001), while there were no differences among the three negative faces (*p*s > 0.999). Moreover, no significant difference in arousal was found [*F*(3, 45) = 1.68, *p* = 0.185, η*_*p*_*^2^ = 0.101) (M(SD): happy 5.71(1.11), angry 6.44(1.47), sad 5.67(1.37), and fearful 6.38(1.00)].

#### Procedure

**FIGURE 1:**
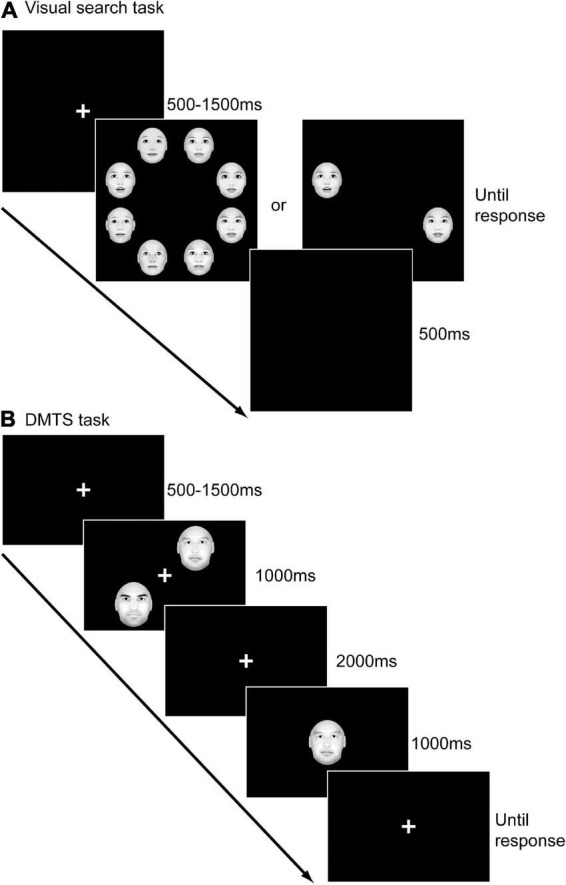
The experimental procedures of the visual search task **(A)** and the delayed-match-to-sample (DMTS) task **(B)**.

Participants sat in a quiet room during the experiment while visual stimuli were presented on a 17-inch liquid crystal display on a Lenovo desktop with a resolution of 1,600 × 900 and a refresh rate of 60 Hz (75). The computer screen was placed 60 cm in front of the participants. As Figure 1A illustrated, each trial of the task began with a white fixation cross presented in the center of the black screen for a random period of 500∼1,500 ms. Afterward, an array of eight faces or two faces appeared until a response was made. Participants were asked to press one key (F) if they found the target (an emotional face) among the distractors (neutral faces) and another key (J) if they didn’t find the target as quickly and accurately as possible. After the response, a blank screen was presented for 500 ms. In total, eight blocks, consisting of 512 trials, were included in this experiment. In four blocks, participants were asked to find the target from seven distractors (set size = 8; high load condition). In the other four blocks, they were asked to find the target from one distractor (set size = 2; low load condition). In each block, the target emotion was fixed. 48 out of 64 trials in each block contained a target, while the other 16 trials did not. The sequence of the eight blocks was randomized among participants.

#### Design and analysis

The visual search experiment is a 4 (Facial emotion: sad, angry, fearful, and happy) × 2 (Load: high and low) × 2 (Group: CD and non-CD) mixed design. The dependent variables included accuracy rate, reaction time and search slope. First, we excluded outliers of trials defined as RTs outside M ± 3SD. Then, we calculated the search slope for each emotional target. The search slope is the slope of the linear fitting line with reaction time versus set size (76). It is an indicator of search efficiency, as a smaller search slope indicates more effective searching and a higher sensitivity to the target. Next, two 4 (emotion) × 2 (load) × 2 (group) repeated measures ANOVAs were conducted on the accuracy and the reaction time respectively, while a 4 (emotion) × 2 (group) repeated measures ANOVA was conducted on the search slope.

### Delayed-match-to-sample task

#### Stimuli

Faces were also selected from the CAPS, including 24 sad, 24 angry, 24 happy, 24 fearful, and 24 neutral faces. Each emotional category included 12 male and 12 female faces. All images were processed similar to the visual search task, except that the image size was set to 185 × 200 pixels. Repeated measures ANOVA showed that the valences were different among emotional categories [*F*(4, 92) = 303.14, *p* < 0.001, η*_*p*_*^2^ = 0.929). *Post-hoc* tests suggested that the valence of happy faces (*M* = 6.46, *SD* = 0.59) was significantly higher than angry (*M* = 2.70, SD = 0.43), sad (*M* = 2.97, *SD* = 0.67), and fearful (*M* = 2.79, *SD* = 0.39) faces (ps < 0.001), while there were no differences among the negative emotions (*p*s > 0.843). Moreover, no significant difference in arousal was found [*F*(3, 45) = 1.68, *p* = 0.185,η*_*p*_*^2^ = 0.101) (M(SD): happy 5.51(1.23), angry 6.18(1.25), sad 5.64(1.39), and fearful 6.32(1.23)].

#### Procedure

As Figure 1B illustrated, each trial of the task began with a white cross on a black background for a random period of 500∼1,500 ms. Subsequently, two faces (sample) with the same expression appeared for 1,000 ms at two of the locations of upper left, lower left, upper right and lower right. Afterward, a blank screen was presented for 2,000 ms, and participants were asked to maintain the two faces they just saw in their minds. Next, a test face was presented in the center of the screen for 1,000 ms. After the disappearance of the test stimulus, the fixation appeared again until participants made a response. Participants were asked to determine whether the test face was one of the two sample faces. All faces presented in a block had the same emotion. The experiment consisted of five blocks. Each block included 48 trials, in half of which the test face matched the sample face. The sequence of blocks was randomized among participants.

#### Design and analysis

The present experiment is a five (Facial emotion: sad, angry, fearful, happy, and neutral) × 2 (Group: CD and non-CD) mixed design. Accuracy, RT, and indicators in signal detection theory (SDT) were treated as dependent variables. Outliers of trials were first excluded in the same way as the visual search task. Discriminability (d’) and reporting criterion (C) were then calculated based on the SDT. In the present experiment, a signal is defined as the matched trials while noise is defined as the unmatched trials. Hit rate (H) is calculated as the rate of the trials in that participants made a yes response in a matched trial, while false alarm rate (FA) is the rate of the trials in that participants made a yes response in an unmatched trial. Then, the hit rate and the false alarm rate are transformed into Z-scores, respectively [i.e., Z(H) and Z(F)]. d’ and C were calculated as: d’ = Z(H)–Z(F) and C = 0.5 × [Z(H)+Z(F)]. A larger d’ indicates stronger discriminability between signal and noise, which reflects stronger working memory capacity. A larger C implies a stricter reporting criterion, which reflects participants are conservative to report signals. Four 5 (emotion) × 2 (group) repeated measures ANOVAs were conducted on the accuracy, the RT, the d’ and the C.

## Results

### Scale results

Scores of CD screening, ICU, CTQ-SF, AQ, SCS, and MDS in each group were displayed in Table 1. MANOVA revealed that CDs showed higher scores on each dimension of, and the total CD scores compared with non-CDs. However, on the other scales, we did not find any significant difference between groups.

**TABLE 1:** Scale scores in conduct disorder (CD) group and non-CD group.

	Non-CD group (*N* = 35) *M* ± *SD*	CD group (*N* = 35) *M* ± *SD*	*F*	*p*	$\eta_{p}^{2}$
Cruelty to human and animals	0.63 ± 0.69	2.20 ± 1.41	35.078	**<0.001**	0.340
Destruction of property	0.03 ± 0.17	0.34 ± 0.59	9.142	**0.004**	0.119
Deception or theft	0.37 ± 0.73	1.03 ± 0.98	10.048	**0.002**	0.129
Serious violations	0.69 ± 0.76	2.06 ± 0.91	47.190	**<0.001**	0.410
Total CD scores	1.71 ± 1.27	5.63 ± 2.17	84.680	**<0.001**	0.555
CU traits	29.93 ± 7.14	29.23 ± 8.97	0.130	0.720	0.002
Childhood maltreatment	47.90 ± 13.32	45.90 ± 10.63	0.485	0.489	0.007
Aggression	55.03 ± 16.58	56.91 ± 14.92	0.250	0.619	0.004
Self-control	58.60 ± 10.94	56.37 ± 10.21	0.776	0.381	0.011
Moral disengagement	78.57 ± 15.22	79.62 ± 15.95	0.080	0.779	0.001

Bold values indicate p < 0.05.

### Visual search task results

**FIGURE 2:**
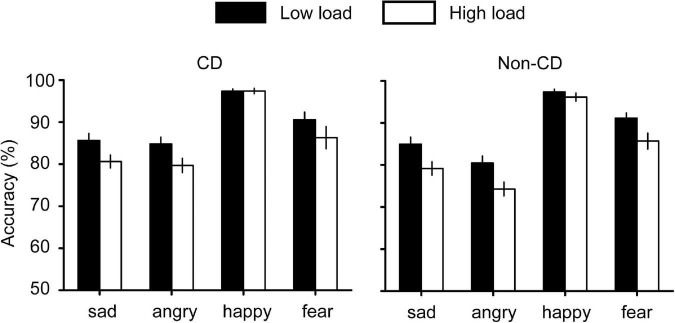
Accuracy results for the visual search task. In low load condition, the search array contains two faces. In high load condition, the search array contains eight faces.

For accuracies (Figure 2), we performed a 4 (emotion) × 2 (load) × 2 (group) repeated measures ANOVA and found a significant interaction effect between emotion and load [*F*(3, 204) = 5.699, *p* = 0.001, η*_*p*_*^2^ = 0.077). A simple effect analysis indicated that the accuracy for sad faces was higher than for angry faces only when the load was high (*p* = 0.043). All other interaction effects were not significant (all *F*s < 2.3, *p*s > 0.08). The main effects of emotion [*F*(3, 204) = 109.839, *p* < 0.001, η*_*p*_*^2^ = 0.618] and load [*F*(1, 68) = 40.587, *p* < 0.001, η*_*p*_*^2^ = 0.374] were significant. However, we did not find any significant interaction or main effect related to the group, indicating that CD may not affect the accuracy of the visual search.

**FIGURE 3:**
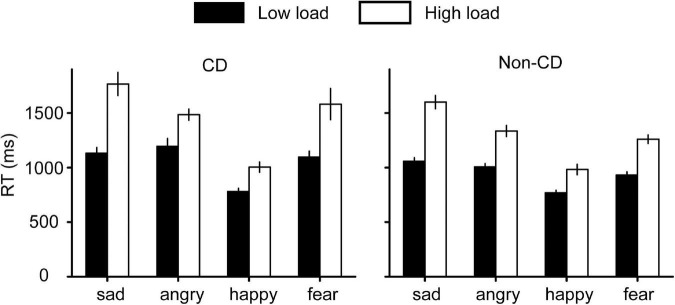
Reaction time (RT) results for the visual search task.

For RTs (Figure 3), a similar 4 (emotion) × 2 (load) × 2 (group) repeated measures ANOVA was conducted. First, the interaction effect between emotion and group was significant [*F*(3, 204) = 3.389, *p* = 0.019, η*_*p*_*^2^ = 0.047]. A simple analysis revealed that RTs for angry and fearful faces were larger in the CD group compared with the non-CD group (angry: *p* = 0.012, fearful: *p* = 0.012), but there were no group differences for sad (*p* = 0.131) and happy faces (*p* = 0.742). Second, the interaction effect between emotion and the load was significant [*F*(3, 204) = 11.178, *p* < 0.001, η*_*p*_*^2^ = 0.141]. A simple effect showed that, when the load was high, the RT for sad faces was longer than that for angry faces (*p* < 0.001), but the trend disappeared when the load was low (*p* > 0.999). Other interaction effects were non-significant (all *F*s < 1.3, *p*s > 0.26). Third, the main effects for emotion [*F*(3, 204) = 69.076, *p* < 0.001, η*_*p*_*^2^ = 0.504], load [*F*(1, 68) = 237.956, *p* < 0.001, η*_*p*_*^2^ = 0.778], and group [*F*(1, 68) = 5.440, *p* = 0.023, η*_*p*_*^2^ = 0.074] were all significant. Taken together, these results indicated attentional deficits in CDs for angry and fearful faces.

For search slopes (Table 2), a 4 (emotion) × 2 (group) repeated measures ANOVA was performed. The interaction effect and the main effect of the group were non-significant. The main effect of emotion was significant [*F*(3, 204) = 11.178, *p* < 0.001, η*_*p*_*^2^ = 0.141]. A *post-hoc* test demonstrated that the slope for sad faces was larger than those for angry (*p* = 0.002) and happy (*p* < 0.001) faces. The slope for happy faces was smaller than that for fearful faces (*p* = 0.026). These results revealed a happy superiority effect in visual search, but CD may not affect the search efficiencies for emotional faces.

**TABLE 2:** The average search slopes in the visual search task.

	Non-CD group (*n* = 35)		CD group (*n* = 35)	
		
Facial expressions	*M*	*SD*	*M*	*SD*
Sad	90.35	48.03	105.77	100.86
Angry	54.66	49.40	48.49	59.09
Happy	35.59	31.38	37.23	27.26
Fearful	54.72	35.98	80.96	119.23

### Delayed-match-to-sample task results

For accuracies (Table 3), a 5 (emotion) × 2 (group) repeated measures ANOVA was performed. The interaction effect and the main effect of the group were non-significant (all *F*s < 0.5, *p*s > 0.83). The main effect of emotion was significant [*F*(4, 260) = 20.456, *p* < 0.001, η*_p_*^2^ = 0.239]. A *post-hoc* test suggested that the accuracies for neutral and happy faces were lower than those for negative affective faces (ps < 0.006). However, there was no difference between neutral and happy faces (*p* > 0.999), nor among three negative affective faces (all *p*s > 0.36).

**TABLE 3:** The average accuracies in delayed-match-to-sample (DMTS) task (%).

	Non-CD group (*n* = 35)		CD group (*n* = 32)	
		
Facial expression	*M*	*SD*	*M*	*SD*
Sad	69.76	13.62	68.93	15.39
Angry	70.70	11.93	70.23	16.92
Happy	64.16	11.34	63.58	13.97
Fearful	72.71	10.86	71.22	17.57
Neutral	61.60	10.65	62.01	13.04

For RTs (Table 4), a similar 5 (emotion) × 2 (group) repeated measures ANOVA was performed. We did not find any significant interaction or main effects (all *F*s < 0.8, *p*s > 0.82).

**TABLE 4:** The average reaction time (RT)s in delayed-match-to-sample (DMTS) task (ms).

	Non-CD group (*n* = 35)		CD group (*n* = 32)	
		
Facial expression	*M*	*SD*	*M*	*SD*
Sad	876.08	234.52	843.04	215.78
Angry	848.72	209.19	845.94	251.63
Happy	899.48	241.67	830.64	243.78
Fearful	832.67	176.67	841.78	191.11
Neutral	861.71	183.85	846.37	281.90

For d’ (Table 5), 5 × 2 repeated measures ANOVA showed a non-significant interaction effect and the main effect of the group (all *F*s < 0.4, *p*s > 0.85). The main effect of emotion was significant [*F*(4, 260) = 18.202, *p* < 0.001, η*_*p*_* = 0.219]. A *post-hoc* test suggested that the d’ for neutral and happy faces were lower than those for negative affective faces (ps < 0.025). However, there was no difference between neutral and happy faces (*p* > 0.999), nor among three negative affective faces (all *p*s > 0.34).

**TABLE 5:** The average discriminability (d’) in delayed-match-to-sample (DMTS) task.

	Non-CD group (*n* = 35)		CD group (*n* = 32)	
		
Facial expression	*M*	*SD*	*M*	*SD*
Sad	1.24	0.87	1.12	0.96
Angry	1.25	0.73	1.31	1.08
Happy	0.87	0.76	0.86	0.88
Fearful	1.39	0.68	1.31	1.10
Neutral	0.71	0.64	0.72	0.78

For C (Table 6), 5 × 2 repeated measures ANOVA revealed that there were no significant interactions or main effects (all *F*s < 1.4, *p*s > 0.25).

**TABLE 6:** The average reporting criterion (C) in delayed-match-to-sample (DMTS) task.

	Non-CD group (*n* = 35)		CD group (*n* = 32)	
		
Facial expression	*M*	*SD*	*M*	*SD*
Sad	0.16	0.52	0.30	0.43
Angry	0.16	0.40	0.34	0.51
Happy	0.13	0.50	0.22	0.65
Fearful	0.18	0.42	0.23	0.48
Neutral	0.09	0.52	0.21	0.57

## Discussion

Facial emotion conveys rich social information and plays an important role in social interaction. The present study explored how CD affects the cognitive processing of emotional faces, including visuospatial attention and visual working memory, among male adolescent delinquents. We introduced a strict experimental control, as the CDs and non-CDs were all adolescent delinquents and lived in the same environment. As a result, the differences between the two groups may largely attribute to the disorder. The results thus provide critical empirical evidence for relevant theories on CD. Specifically, our results mainly support the optimal stimulation/arousal theory, the stimulation-seeking theory and the fearlessness theory, but not the social-cognitive theory.

The core finding of our study is that adolescents with CD searched angry and fearful faces more slowly compared with non-CDs, which was not found on sad and happy faces. These deficiencies were independent of the load, indicating global deficits in attentional orientation to hostile and threatening faces among CDs. It was partly consistent with a previous study which revealed that adolescents with CD fixated less on fearful and sad faces (48). The results support the optimal stimulation/arousal theory (35–37), the stimulation-seeking theory (38, 39) and the fearlessness theory (40, 41). According to these theories, individuals with CD may experience less fear and have a lower level of physical arousal. As a result, they would show less response to affective faces. Physiological measurements revealed that individuals with CD showed reduced baseline or resting heart rate, skin conductance and electrodermal activity (77, 78), reduced amplitudes of startle reflex (79–81), and reduced response in the hypothalamus-pituitary-adrenal axis (77, 82–84). In addition, individuals with CD also showed deficits in fear conditioning, suggesting that they are fearless (80, 85). Angry and fearful faces convey hostility or threatening signals, and thus may draw attention automatically. For example, in some studies, people found a larger attentional bias toward fearful faces relative to happy and sad faces, even the fearful face was unaware (86–91). Similarly, other studies also showed attentional bias toward angry faces (49–53). Correspondingly, larger P1 amplitude was found elicited by angry faces relative to happy and sad faces (92–95). In the present study, we showed that adolescents with CD might not be as sensitive as the non-CDs to experience hostile or threatening signals. It may be a crucial reason for their aggressive behaviors.

Our study provided a strict control on the environmental variables, as both groups were selected from the same facilities, and all participants were delinquents. It’s very important to control the living environment between groups, for the environment and partnership may largely affect the behavior and cognition of an individual (15, 96–108). From the scale results, we found that the two groups were not significantly different from each other in CU traits, childhood maltreatment, aggression, self-control and moral disengagement, indicating that the groups were well matched for aspects other than CD. Therefore, our results provide convincing evidence and make us reconsider previous theories and findings. For example, a previous study adopting the dot-probe paradigm found that adolescents with CD showed attentional avoidance of angry, fearful and happy faces compared to typically developing adolescents recruited from schools and colleges (47). Another study revealed that youths with CD showed impairments in recognition of anger, disgust, fear, happiness, sadness and surprise, compared with typically developing controls (109, 110). One possible reason for the difference between these results and ours might be the influence of peers and the environment. Further studies are required to examine these influential factors more detailed.

Compared with attentional processing, the working memory processing was found hardly affected by CD. We only found higher accuracies and discriminability for negative affective faces, indicating a general negative mnemonic bias among adolescent delinquents. It should be noted that this mnemonic bias could not be attributed to higher sensitivity to these emotional faces, as we showed a happy superiority effect in the visual search task. These results also indicated that the attentional processing and mnemonic processing on emotional faces may not share the same underlying mechanisms, and adolescent delinquents may preserve negative faces more stably and accurately. A larger number of previous studies revealed that, compared with happy and neutral faces, angry faces significantly improved the working memory capacity for facial identity (111–115), and the working memory sensitivity for fearful faces was higher than that for neutral faces (116). Working memory is an ability that preserves and updates information in a short period of time (117–119). The stronger working memory processing on negative emotional faces among adolescent delinquents may imply difficulty in refreshing negative information, and thus cause aggressive behaviors. Nevertheless, more evidence is needed to further elucidate the relationship between working memory processing and aggression.

Finally, we noticed that there were several limitations in the present study. First, we did not include a typically developing group. As we discussed above, the difference between delinquents and typically developing individuals may largely attribute to the difference in environment and partnership, and thus the comparison does not help understand the effect of CD. Further study concerning the effect on the environment may include such a group to provide more information on the development of CD. Second, we only investigated the cognitive processing with two typical tasks. Although these tasks are pervasively adopted to assess cognitive processing, there are also limitations in these tasks. For example, visual search task could mainly examine the attentional bias but not attentional disengagement. Regarding working memory, DMTS may mainly assess the maintaining of working memory, which is a part of the common working memory ability (120). Future studies may adopt n-back task to examine the updating process of emotional faces (121, 122). In addition, memory load may impact the performance (123–125). In the present study, we set the memory load at a moderate level to avoid any ceiling effect and floor effect. Nevertheless, working memory load should be manipulated as an independent variable in future studies. In summary, other tasks and paradigms may be further included to reveal the effect of CD on more aspects of cognitive processing. Third, only male adolescents were recruited in the present study, as there were few female delinquents in the local facilities. Further studies may focus on the effect of CD on the cognitive processing among female adolescents and reveal common and different mechanisms between male and female delinquents.

## Conclusion

Male adolescent delinquents with CD showed deficits in attentional orientation to hostile and threatening faces (e.g., angry and fearful faces), partly supporting the optimal stimulation/arousal theory, the stimulation-seeking theory and the fearlessness theory.

## Data availability statement

The raw data supporting the conclusions of this article will be made available by the authors, without undue reservation.

## Ethics statement

The studies involving human participants were reviewed and approved by Ethical Committee of Human Research at Zunyi Medical University. Written informed consent to participate in this study was provided by the participants’ legal guardian/next of kin.

## Author contributions

HK: conception, design, acquisition of data, analysis of data, interpretation of data, funding acquisition, writing-original draft, and writing-review and editing. WL: conception, design, data acquisition, data analysis, data interpretation, and writing-review and editing. XL: conception, design, and analysis of data. YY and MX: acquisition of data and writing-review and editing. BS: acquisition of data. QX: conception, design, analysis of data, funding acquisition, and writing-review and editing. TB: conception, design, analysis of data, interpretation of data, funding acquisition, and writing-review and editing. All authors contributed to the article and approved the submitted version.
